# Granulocytic myeloid-derived suppressor cells suppress virus-specific CD8^+^ T cell responses during acute Friend retrovirus infection

**DOI:** 10.1186/s12977-017-0364-3

**Published:** 2017-08-23

**Authors:** Malgorzata Drabczyk-Pluta, Tanja Werner, Daniel Hoffmann, Qibin Leng, Lieping Chen, Ulf Dittmer, Gennadiy Zelinskyy

**Affiliations:** 1 Institute of Virology, University Hospital Essen, University of Duisburg-Essen, Hufelandstraße 55, 45147 Essen, Germany; 2 Research Group Bioinformatics, Faculty of Biology, University of Duisburg-Essen, Essen, Germany; 3 Key Laboratory of Molecular Virology and Immunology, Institute Pasteur of Shanghai, Institutes for Biological Sciences, Chinese Academy of Sciences, Shanghai, China; 4 Department of Immunobiology, Yale School of Medicine, Yale University, New Haven, CT USA

**Keywords:** MDSC, CD8^+^ T cells, Friend virus, PD-L1, Arginase

## Abstract

**Background:**

Myeloid-derived suppressor cells (MDSCs) can suppress T cell responses in several different diseases. Previously these suppressive cells were observed to expand in HIV patients and in a mouse retrovirus model, yet their suppressive effect on virus-specific CD8^+^ T cells in vitro and in vivo has not been characterized thus far.

**Results:**

We used the Friend retrovirus (FV) model to demonstrate that MDSCs expand and become activated during the late phase of acute FV infection. Only the subpopulation of granulocytic MDSCs (gMDSCs) but not monocytic MDSC suppressed virus-specific CD8+ T cell proliferation and function in vitro. gMDSCs expressed arginase 1, high levels of the inhibitory ligand PD-L1 and the ATP dephosphorylating enzyme CD39 on the cell surface upon infection. All three molecules were involved in the suppressive effect of the gMDSCs in vitro. MDSC depletion experiments in FV-infected mice revealed that they restrict virus-specific CD8^+^ T cell responses and thus affect the immune control of chronic retroviruses in vivo.

**Conclusions:**

Our study demonstrates that MDSCs become activated and expand during the acute phase of retrovirus infection. Their suppressive activity on virus-specific CD8+ T cells may contribute to T cell dysfunction and the development of chronic infection.

## Background

 Subpopulations of myeloid cells are part of the innate immune system and simultaneously these cells are of crucial importance for the development of adaptive immune responses. More than 30 years ago different research groups detected an accumulation of myeloid cells in growing tumors [1, 2]. Later the suppressive function and heterogeneity of these natural suppressor cells, as they were first called, was observed. It was shown that most of these cells have phenotypic and morphologic similarities to immature monocytes and granulocytes and are derived from similar myeloid precursors [3, 4]. These cells were therefore defined as myeloid-derived suppressor cells (MDSC) and divided into the subpopulations monocytic mMDSCs and granulocytic gMDSCs [4]. A hallmark of MDSCs is that they efficiently suppress effector T cell responses, including T cell proliferation and functional properties. Heterogeneity of MDSCs was observed in terms of the functional mechanisms utilized by MDSCs to suppress T lymphocytes. MDSCs can exploit the metabolism of l-arginine by arginase 1 (Arg1) [5], expression of inducible nitric oxide synthetase (iNOS) [5], and/or production of reactive oxygen species (ROS) [6] as main immunosuppressive tools. The production of inhibitory cytokines, the expression of ligands for inhibitory receptors on lymphocytes, and the expression of the surface enzymes CD39 (Ectonucleoside triphosphate diphosphohydrolase-1) and CD73 (Ecto-5′nucleotidase) [7, 8], regulating the metabolism of Adenosine Triphosphate were also described for subpopulations of MDSCs [9–15]. Thus MDSCs are cells with a high suppressive potential, which may simultaneously use diverse inhibitory mechanisms to inhibit T cell responses.

An activation and expansion of MDSCs was also observed during acute and chronic viral infections [16]. High numbers of MDSCs were found in patients infected with HBV [17–20] and HCV [21, 22]. Also HIV-infected patients [23, 24] have an enhanced number of mMDSCs and gMDSCs in the blood. In the murine AIDS model based on a LP-BM5 retrovirus infection of mice, Green and colleagues showed that expanded mMDSCs suppressed B cell activity and polyclonal T cell responses [25–27]. mMDSCs also influenced regulatory T cell responses in this model [28]. The data from HIV patients and from the murine LP-BM5 model demonstrate the negative influence of MDSCs on adaptive anti-retroviral immune responses. However, the suppressive effects of MDSCs on virus-specific cytotoxic CD8^+^ T cell responses was not analyzed and the role of MDSCs for the establishment of a chronic retroviral infection remains elusive. These important questions have been addressed in the current study using the Friend virus (FV) mouse model.

FV is an oncogenic retroviral complex that can induce erythroleukemia in susceptible mice. However, resistant mouse strains, like the C57/Bl6 mice used in this study, mount a potent anti-viral immune response during the acute phase of infection that prevents the onset of leukemia [29]. Despite this efficient initial anti-viral immunity, FV escapes from T cell mediated immune control and establishes a chronic infection [30, 31]. Cytotoxic CD8^+^ T lymphocytes (CTL) are crucial for controlling FV replication during the acute phase of infection. However, during chronic FV infection, similar to the human immunodeficiency virus (HIV) and hepatitis C virus (HCV) infections, virus-specific CD8^+^ T cells become functionally exhausted. This exhaustion most likely contributes to the inability of the host to eliminate cells infected with the pathogen [32, 33]. Thus, our aim was to define whether MDSCs contribute to the T cell exhaustion that develops during chronic FV infection.

The current study shows the expansion of gMDSCs and mMDSCs during the late phase of acute FV infection. In contrast to the murine AIDS retrovirus model, the suppressive effect on virus-specific CD8^+^ T cells was mainly mediated by gMDSCs. Results from in vivo MDSCs depletion experiments showed that these cells contribute to the development of chronic FV infection and hence, may be an important target for immunomodulatory therapies of acute or chronic viral infections.

## Results

### Myeloid-derived suppressor cells expand during FV infection

MDSCs were shown to play a role in the suppression of immune responses not only in cancer, but also in various infectious diseases such as LCMV, HCV, HBV, and HIV [34–37]. However their exact role in retroviral infections is still elusive.

**Fig. 1:**
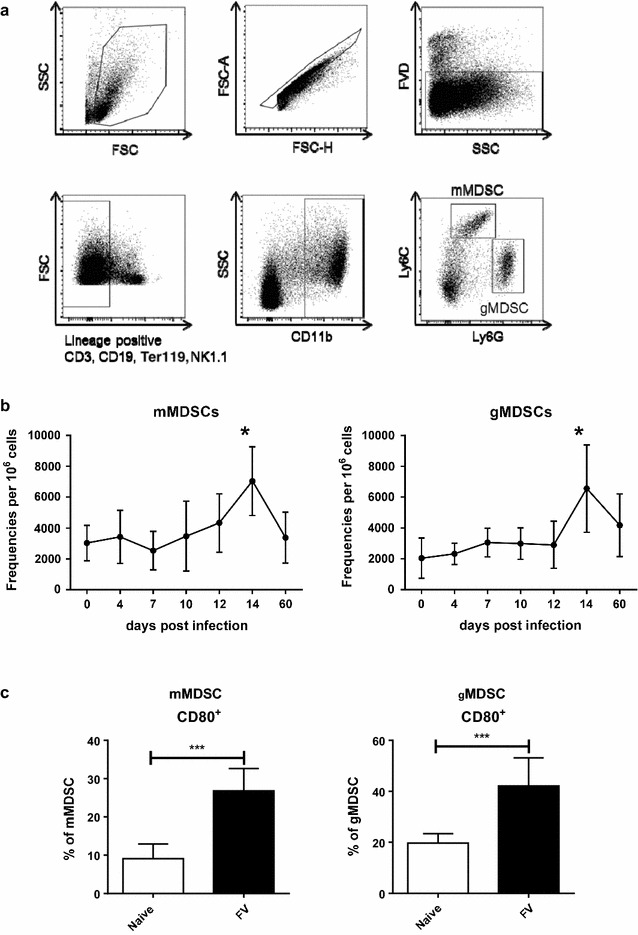
MDSCs expand during acute FV infection. B6 mice were infected i.v. with 20,000 SFFU of FV or left un-infected, and MDSC numbers were measured using flow cytometry. The total spleen cells were analyzed at various time points post infection. **a** The numbers of Ly6G^high^ Ly6C^low^ and **b** Ly6G^−^ Ly6C^high^ per 1 × 10^6^ live splenocytes and the frequencies of **c** CD80 expression on the surface of Ly6G^high^ Ly6C^low^ and Ly6G^−^ Ly6C^high^ cells are displayed. At least 7 mice per group from five independent experiments were analyzed. *Bars* represent means with SD. For statistical analysis a Dunn’s test with the Benjamini–Hochberg correction for multiple testing (**b**) and an unpaired t test (**c**) were performed (*<0.05; **<0.005)

In order to characterize MDSCs during FV infection, B6 mice were infected and the kinetic of the MDSC response was determined. MDSCs were characterized according to recommendations for the phenotypic definition of these cells [38–40] CD11b+ cells were gated from live, single-cell splenocytes, which were negative for CD3, CD19, NK1.1, and Ter119 lineage markers. These CD11b+ myeloid cells were divided according to their expression of Ly6C and Ly6G in monocytic (mMDSC, Ly6G-Ly6C^high^) and granulocytic (gMDSC, Ly6G^high^ Ly6C^low^) MDSCs (Fig. 1a). mMDSCs and gMDSCs were detectible in naïve mice, but their frequencies were below 0.3% per one million nucleated splenocytes. This number of MDSCs was stable during early FV infection until day 10. At day 14 after infection, the population of mMDSCs and gMDSCs expanded significantly. The expansion of both MDSC subpopulations peaked on day 14 post infection in the spleen (Fig. 1b) and the frequency of these cells reached more than 6000 per one million spleen cells. During the chronic phase of FV infection, the numbers of both the granulocytic and monocytic MDSCs remained slightly elevated (mean number 3200 mMDSCs and 3100 gMDSCs per one million spleen cells) in comparison to non-infected mice (mean number 2700 mMDSCs and 2500 gMDSCs per one million spleen cells) (Fig. 1b). However these differences were not significant. These data indicate that the populations of mMDSCs and gMDSCs mainly expanded at day 14 after FV infection, a time point that is concomitant with the onset of the suppression of virus-specific effector CD8^+^ T cell responses [41, 42].

CD80 is a member of the B7 family and is a stimulatory or inhibitory molecule of T cell activation. It is a ligand for two receptors: CTLA-4 and CD28. While CD28 induces T cell activation, CTLA-4 mediates T cell suppression [43]. Activated MDSCs isolated from tumor bearing mice [44] and from cancer patients [45] show a significantly enhanced expression of CD80, defining CD80 as activation marker for MDSCs. Approximately 10% of the mMDSCs and 20% of the gMDSCs from naïve mice expressed CD80 (Fig. 1c). At day 14 post infection a mean of 25% of the mMDSCs and 43% of the gMDSCs expressed CD80 (Fig. 1c). These data demonstrate that MDSCs expanded and became activated in the late phase of acute FV infection.

### gMDSC suppress virus-specific CD8^+^ T cell responses in vitro

**Fig. 2:**
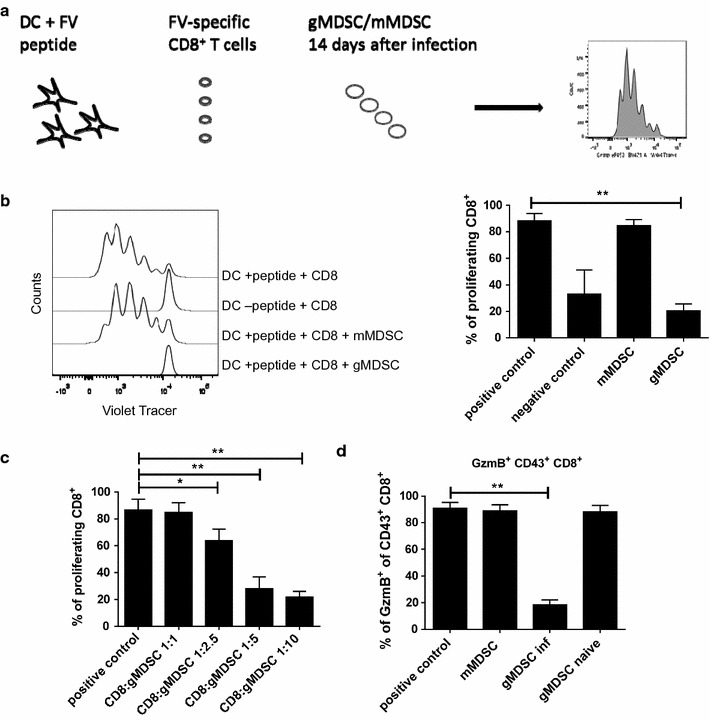
Granulocytic myeloid-derived suppressor cells inhibited CD8^+^ T cell proliferation. CD8^+^ T cells isolated from DbGagL TCR transgene mice were incubated with dendritic cells loaded with MHC class I-restricted FV-specific CD8^+^ T cell epitope peptide and co-incubated with either gMDSCs or mMDSCs (**a**). Representative histograms and percentages of CD8^+^ T cells after co-incubation with or without either gMDSC or mMDSCs (in relation 1 CD8^+^ to 10 MDSCs) from FV-infected mice are shown (**b**). CD8^+^ T cell proliferation was measured after co-incubation with different effector ratios of gMDSCs to CD8^+^ target cells (**c**). Frequencies of GzmB expressing CD43^+^CD8^+^ cells after incubation of CD8^+^ cells with gMDSCs or mMDSCs from FV-infected mice are shown (**d**). CD8^+^ T cells incubated with peptide loaded DC serve as a positive control, CD8^+^ T cells incubated with non-loaded DC serve as a negative control. At least three independent experiments were analyzed. Bars represent the mean with SD. For statistical analysis, an ANOVA multiple comparison test was carried out with the group of naïve mice as a reference (*<0.05; ***<0.0005). For statistical analysis a Dunn’s test with the Benjamini–Hochberg correction for multiple testing was performed (*<0.05; **<0.005)

MDSCs display a certain phenotype, but their main characteristic is their suppressive activity against T cell responses. We therefore analyzed whether FV-induced MDSCs can suppress FV-specific effector CD8^+^ T cells in an in vitro model. To achieve this goal, a FV-specific CD8^+^ T cell proliferation assay was established. Bone marrow derived dendritic cells were incubated with Violet Cell tracer labeled virus-specific CD8^+^ T cells isolated from TCR transgenic mice, of which more than 90% of the CD8^+^ T cells contain a TCR specific for the DbGagL FV epitope [46, 47]. The DCs were loaded with the DbGagL epitope peptide to induce a virus-specific proliferation of the CD8^+^ T cells. mMDSCs and gMDSCs were isolated from FV-infected mice at 14 dpi, according to their expression of Ly6C and Ly6G respectively. In order to determine the suppressive effect of these subpopulations on the virus-specific CD8^+^ T cell response, enriched mMDSCs or gMDSCs were added in a 10:1 MDSC to CD8^+^ T cell (E:T) ratio. After 3 days of culture, CD8^+^ T cell proliferation and effector molecule granzyme B (GzmB) expression were analyzed. At this time point an average of 90% of the CD8^+^ T cells had undergone at least one cell division. Interestingly, this CD8^+^ T cell proliferation was only suppressed by gMDSCs, but not by mMDSCs (Fig. 2b). To clarify the suppressive potential of gMDSCs, these cells were added to target CD8^+^ T cells in different ratios (1:1, 2.5:1, 5:1, 10:1 gMDSCs to CD8^+^ T cells) (Fig. 2c). An increasing reduction of CD8^+^ T cell proliferation was observed at ratios of gMDSC to CD8+ T cells of 2.5:1 and higher. Thus, the gMDSCs mediated suppression was cell number dependent.

Additionally, the GzmB expression in activated CD8^+^ T cells was measured. An average of 90% of all CD8^+^ T cells in the cultures produced GzmB. This expression of GzmB was also diminished by gMDSCs, but not by mMDSCs co-incubation (Fig. 2d). At an effector target ratio of 1:10 gMDSCs reduced the percentage of GzmB^+^ CD8^+^ T cells to a mean of below 20% (Fig. 2d). gMDSCs from naïve mice did not suppress the proliferation or the GzmB production of FV-specific CD8^+^ T cells, indicating that the suppressive activity of this MDSC subpopulation was induced by FV infection.

These data demonstrate that gMDSC but not mMDSCs from FV-infected mice were able to suppress virus-specific CD8^+^ T cell responses in vitro.

### Molecules involved in FV-induced T cell suppression by gMDSCs

**Fig. 3:**
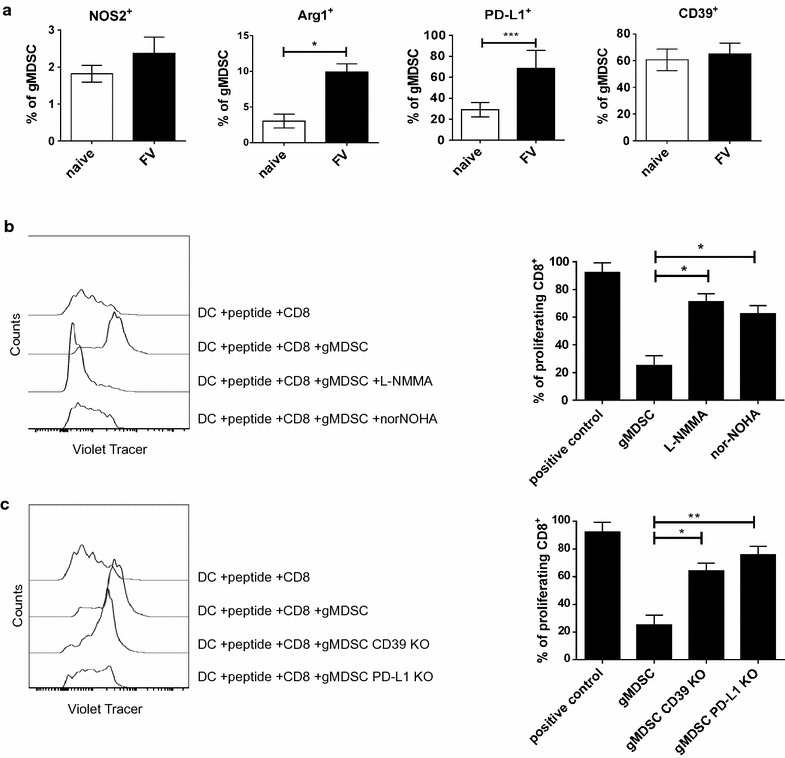
Mechanisms of MDSC mediated suppression. B6 mice were infected i.v. with 20,000 SFFU of FV or left un-infected and the expression of Arg1 and NOS2 in the MDSCs was measured using flow cytometry for spleen cells at day 14 post infection. Frequencies of NOS2, Arg1, PD-L1, and CD39 expressing Ly6G^high^ Ly6C^low^ cells in the spleen are displayed (**a**). At least five mice per group from three independent experiments were analyzed. *Bars* represent the mean with SD. For statistical analysis an unpaired t test was performed (*<0.05; ***<0.0005). **b** CD8^+^ T cells isolated from DbGagL TCR transgenic mice were incubated with dendritic cells loaded with MHC class I-restricted FV-specific CD8^+^ T cell epitope peptide and co-incubated with gMDSCs in the ratio of 1 CD8^+^ to 10 gMDSCs with or without the addition of L-NMMA or nor-NOHA. Representative histograms and percentages of proliferating CD8^+^ T cell proliferation after co-incubation with or without gMDSCs in the presence or absence of L-NMMA/nor-NOHA are shown. At least three independent experiments were analyzed. *Bars* represent the mean with SD. **c** CD8^+^ T cells isolated from DbGagL TCR transgenic mice were incubated with dendritic cells loaded with MHC class I-restricted FV-specific CD8^+^ T cell epitope peptide and co-incubated with gMDSCs isolated from CD39 KO mice or from PD-L1 KO mice. Representative histograms and percentages of proliferating CD8^+^ T cells after co-incubation with or without gMDSCs isolated from B6, CD39 KO and PD-L1 KO FV infected mice are shown. At least three independent experiments were analyzed. The *bars* represent the mean with SD. For statistical analysis a Dunn’s test with the Benjamini–Hochberg correction for multiple testing was performed (*<0.05; **<0.005)

It was previously shown that functional MDSCs express different molecules that are associated with their suppressive activity [13, 48–52]. Therefore, NOS2, Arg1, PD-L1, and CD39 expression on the suppressive gMDSCs were analyzed at the peak of their expansion after FV infection (14 dpi). Arg1 was of special interest, an enzyme converting ι-arginine to urea and ι-ornithine, followed by NOS2, an enzyme further synthetizing nitric oxide (NO) and ι-citrulline [14, 48, 51, 53]. Very low frequencies of gMDSCs from naïve animals expressed intracellular NOS2, which did not significantly increase after FV infection (Fig. 3a). On the contrary, the percentage of Arg1 expressing gMDSCs increased during FV infection from a mean of approximately 3% in naïve to 10% in infected mice (Fig. 3a). 30% of the naïve gMDSCs were PD-L1^+^, which increased to about 70% after FV infection (Fig. 3a). In contrast, already 60% of the gMDSCs expressed CD39 prior to infection and this expression level did not significantly change after FV inoculation (Fig. 3a).

In order to investigate whether CD8^+^ T cell suppression was arginase or NO dependent, the previously described proliferation assay was used. In this assay nor-NOHA, which is a pan arginase inhibitor, or L-NMMA, a pan NO inhibitor, were added to the cultures. After administration of the arginase inhibitor norNOHA, we observed an approximately 2.5 times higher percentage of proliferating CD8^+^ T cells in the presence of gMDSCs in comparison to the untreated control (Fig. 3b). Administration of the NO inhibitor L-NMMA resulted in an almost 3 times higher percentage of proliferating CD8^+^ T cells compared to the untreated group (Fig. 3b). The suppression of T cell responses by gMDSCs was not reversed after the control molecule D-NMMA was added (data not shown). These data suggest that ι-arginine metabolism is at least one mechanism of gMDSCs mediated suppression of virus-specific T cell proliferation in vitro.

To analyze the role of CD39 and PD-L1 in the T cell suppression by gMDSCs, the previously described proliferation assay was again used. For these experiments, gMDSCs were isolated from FV-infected CD39 or PD-L1 knockout mice. gMDSCs lacking PD-L1 were not able to efficiently suppress CD8^+^ T cell proliferation in vitro (76% of all CD8^+^ T cells proliferated, very similar to the positive control) (Fig. 3c). After incubation of gMDSCs isolated from CD39 KO mice with CD8^+^ T cells, 64% of the CD8^+^ T cells had undergone cell division, which was almost 2.5 higher as in the control group with gMDSCs from wild type mice (Fig. 3c). Interestingly, full suppression of T cell responses was observed when gMDSCs were isolated from CD73 (5′-nucleotidase (5′-NT)) knockout mice (data not shown). These data suggest a significant role of PD-L1 expressed and adenosine metabolism in gMDSC mediated suppression.

### Selective depletion of MDSCs in FV-infected mice

After characterizing the MDSC activity in vitro it was of interest to confirm their suppressive potential in vivo during an ongoing FV infection. Different methods to eliminate, block or suppress MDSCs have been described, including antibody treatment (αGr1, αLy6G), directly acting drugs (5-Fluorouracil (5FU), Silendafil, doxorubicine) and drugs which were described to maturate MDSCs (ATRA, CpG) [54].

**Fig. 4:**
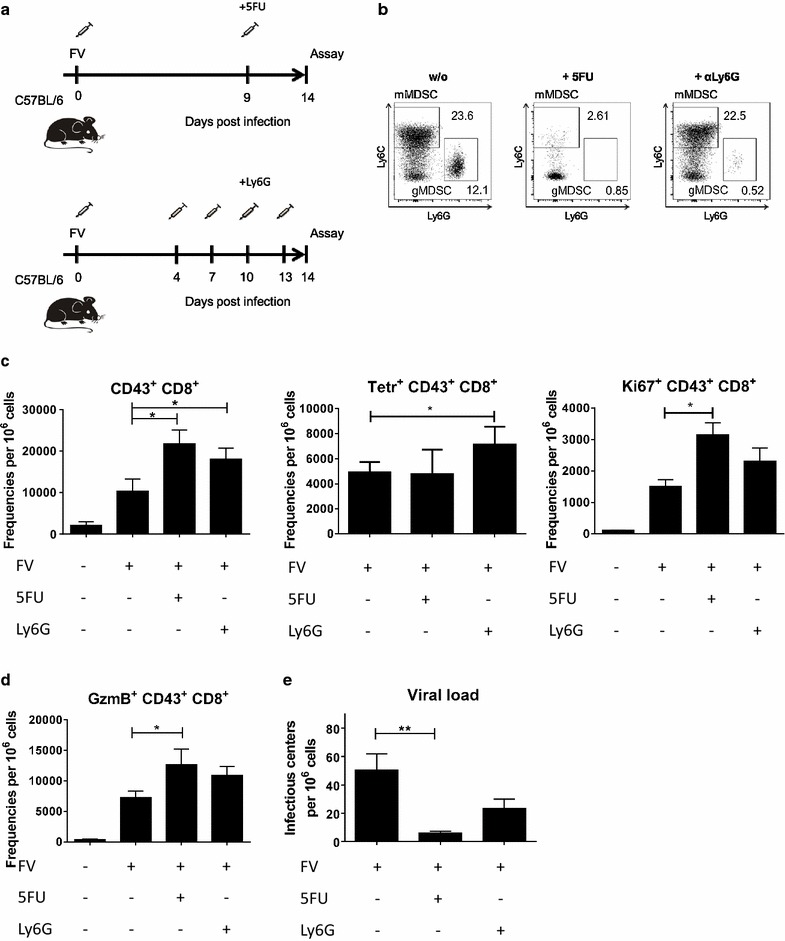
Depletion of MDSCs. B6 mice were infected i.v. with 20,000 SFFU of FV, and/or treated with 5FU or αLy6G. CD8^+^ numbers were measured using flow cytometry and viral loads were estimated for spleen cells at 14 days post infection. Experimental design of the MDSC depletion by administration of 5FU or αLy6G (**a**). **b** Representative dot plots of MDSC during FV infection after administration of 5FU, αLy6G or untreated. **c** The numbers of CD43^+^ CD8^+^ T cells, the numbers of FV-D^b^gagL class I tetramers positives CD8^+^CD43^+^, and the numbers of proliferating Ki67^+^CD43^+^CD8^+^ T cells per 1 × 10^6^ live cells in the spleen are displayed. **d** The numbers of CD43^+^ CD8^+^ T cells expressing GzmB per 1 × 10^6^ live cells in the spleen are displayed. **e** The numbers of viral loads (infectious centers per 1 × 10^6^ cells) in the spleen are displayed. At least five mice per group from three independent experiments were analyzed. Bars represent the mean with SD. For statistical analysis a Dunn’s test with the Benjamini–Hochberg correction for multiple testing was performed (*<0.05; **<0.005)

In order to investigate the role of MDSCs during FV infection in living mice either gMDSCs alone or both the gMDSC and mMDSC populations were depleted. By a single administration of 5FU on day 9 post FV infection (Fig. 4a), both MDSC populations were efficiently depleted in the spleen (Fig. 4b). The pyrimidine analog 5FU is a cytostatic drug with MDSCs specific cytotoxicity. Selective activity of 5FU on MDSC was shown by Vincent et al. [55], and other immune cells were not affected by the drug. We confirmed these findings by staining for all other main immune cell subsets after 5FU injection in naïve mice (data not shown). The second approach, the i.p. administration of an αLy6G antibody, selectively depleted gMDSCs (Fig. 4b). αLy6G antibody was administered four times every third day between day 5 and 13 post FV infection (Fig. 4a). First, we analyzed the CD8^+^ T cell response in FV-infected MDSC depleted mice in the spleen. It was of interest to discover whether the population of FV-specific effector CD8^+^ T cells was influenced by MDSCs in vivo. CD43^+^, a sialoglycoprotein, expressed on the cell surface of T lymphocytes, is a part of the receptor-ligand complex and required for T cell activation [56, 57]. Naïve CD8^+^ T cells do not express CD43^+^, but CD43^+^ becomes highly up-regulated on antigen specific effector CD8^+^ T cells [56]. FV induced CD8^+^ T cells express high levels of CD43 on the cell surface [58]. Interestingly, after the depletion of all MDSCs by 5FU, the frequency of CD43^+^ CD8^+^ T cells increased significantly in comparison to only FV-infected mice (Fig. 4c). After the gMDSC depletion using αLy6G antibodies, also significantly higher frequencies of activated CD8^+^ T cells were observed

Next, it was investigated whether the expanded population of activated CD8^+^ T cells was specific for the immunodominant epitope of FV. With the use of FV-D^b^gagL class I tetramers it was possible to assess that the administration of 5FU did not result in increased percentages of D^b^gagL-specific effector CD8^+^ T cells. However, the depletion of gMDSCs with αLy6G antibodies significantly increased the frequency of tetramer^+^ CD8^+^ T cells, suggesting that mainly gMDSCs act on antigen-specific T cells.

To determine whether the expansion of CD8^+^ T cells after MDSC depletion was driven by increased CD8^+^ T cell proliferation, the intranuclear expression of Ki67, a nuclear protein involved in cell proliferation [59], was analyzed. After 5FU administration significantly more CD43^+^ CD8^+^ cells expressed Ki67 compared to the infected control group. In gMDSC depleted mice also enhanced numbers of proliferating CD8^+^ T cells were detected. However, the difference to control mice was not statistically significant (Fig. 4c).

In order to characterize the effector functions of the expanded CD8^+^ T cells after MDSC depletion, we stained for intracellular GzmB expression. In both groups of 5FU as well as αLy6G treated mice, frequencies of GzmB^+^ CD8^+^ T cells were increased in comparison to control mice, but only the difference after 5FU treatment was statistically significant (Fig. 4c). Thus, the in vivo data from MDSC depletion experiments showed that the expansion of effector CD8^+^ T cells and their differentiation into potentially cytotoxic GzmB producing CTLs were influenced by MDSC during FV infection. In particular, gMDSCs regulated the magnitude of the CD8+ T cell response against the immunodominant CD8 epitope of FV. This effect was also seen in our in vitro suppression assays where the peptide for the FV immunodominant epitope was used.

Since CD8^+^ T cells are necessary for controlling FV replication during acute infection [41], it was important to determine whether MDSC depletion affects viral loads. To answer this question, an infectious center assay was performed at 14 dpi. After depletion of all MDSCs with 5FU, an approximately tenfold reduction in spleen viral loads was observed. After the specific depletion of gMDSCs a more than twofold reduction was found, which was not statistically significant mainly due to the variations within the group of untreated control animals (Fig. 4e). These data suggest that MDSC-mediated suppression of CD8^+^ T cell responses affected the elimination of FV-infected cells in vivo. They also imply that in FV infected mice both MDSC populations may contribute to the suppression of T cell responses.

## Discussion

Myeloid-derived suppressor cells play an important role in regulating immune responses. Their influence may be beneficial, as they limit immunity to prevent tissue damage via T cell cytotoxicity. On the other hand, they may be suppressing immune responses against viral infections, which results in an incomplete elimination of the virus. The exact mechanisms involved in the control of viral immunity by MDSCs are not fully understood. Therefore, it is of importance to define the detailed functions of MDSCs in viral infections.

In this study (Fig. 1a) we defined MDSCs by using antibodies against CD11b^+^, Ly6G^+^ and Ly6C^+^ [38]. It was also of importance to distinguish between MDSC subpopulations, since it has been shown that each subpopulation possesses distinct functions. The phenotype of MDSCs associated with the activation and with functionality of these cells was also characterized by the expression of PD-L1, CD39, CD80, as well as the functional markers Arg1 and NOS2 (Fig. 3a). The described strategy allowed for a precise differentiation of the MDSC subpopulations in mice. Additionally, to correctly define the MDSC populations it was important to assess their suppressive activity as well as the biochemical and molecular markers described for MDSCs. Various studies have used different strategies for the phenotypic definition of MDSCs [60–63]. These differences make it difficult to compare results from theseinvestigations. Recently, attempts to standardize the definition of MDSC phenotypes in different species have been made [38, 64]. The current study strictly followed the state of the art recommendations ensuring that the latest standards of MDSC detection were used.

Expansion of MDSCs was previously observed in various experimental models. Recent studies suggest that MDSC may even serve as a prognostic marker for disease progression in various cancer models as well as during viral infections. Here, we demonstrate the expansion and activation of MDSC during an acute retroviral infection of mice. Elevation of MDSC numbers was also observed in blood of chronic HCV patients [21, 65, 66], as well as during human [17, 19, 20, 67] and mouse HBV infection [36, 68]. The total MDSC population as well as the gMDSC subpopulation were shown to increase in numbers during HIV infection [24, 52, 65].

In some of these studies the suppressive effect of MDSCs on T cell responses in vitro was demonstrated [48, 66, 69]. However, most studies conducted with cells from virus infected patients investigated the whole MDSCs population without distinguishing between different subpopulations [52, 65]. The effects of MDSC subpopulations on immune responses may vary significantly. Our current study demonstrates the suppressive activity of gMDSCs, but not mMDSCs, on virus-specific CD8^+^ T cells in acute FV infection. In contrast to our findings, mMDSCs in a model of murine LP-BM5 retrovirus infection have been observed to preferentially suppress B cell responses, yet also T cell responses were downregulated [25–27]. The different results might be explained by technical differences in the T cell proliferation assays used in both studies. While we stimulated virus-specific T cells with its cognate antigen presented by DCs, a physiological way of inducing T cell proliferation and differentiation, others used a non-specific stimulation of T cells with CD3 and CD28 [25, 61]. It has already been reported that mMDSCs mainly suppress polyclonally activated T cells, as shown in tumor models [70] and infections such as LCMV, HBV, and HCV [36, 71]. On the other hand, a suppression of antigen-specific T cell responses are rather associated with gMDSCs function [72, 73]. Therefore, gMDSCs may have a significant influence on disease progression in various cancers [74], hepatic inflammation, fibrosis [75, 76], and in HIV infection [24].

An activation of MDSCs has been associated with the expression of different functional molecules. CD80, PD-L1, CD73, CD39, CD270, CD62L are just a few markers which were shown to be expressed on the cell surface of MDSCs and were linked to an activation of these cells [13, 14, 51, 77, 78]. In the current study, we analyzed the expression of CD80, as well as PD-L1 and CD39 as cell surface activation markers. An enhanced PD-1 expression on virus-specific CD8^+^ T cells in chronically infected mice was shown to be involved in the development of CD8^+^ T cell exhaustion. AIl FV-infected mice CD8^+^ T cells also up-regulate PD-1, which plays an important role in immune evasion during acute infection [41, 79, 80]. Thus, PD-L1 expressing MDSCs might be a critical cell population inducing T cell exhaustion in chronic viral infections [34, 81]. This possibility was already discussed in various diseases, e.g. multiple melanoma [82, 83] or HIV infection [34]. Bowers et al. [34] illustrated the role of PD-1/PD-L1 signaling in T cell suppression mediated through HIV expanded gMDSC. We confirmed these results in our in vitro analysis of virus-specific CD8^+^ T cell proliferation. gMDSCs used for this study lacked PD-L1, and were not able to suppress CD8^+^ proliferation as efficiently as wild type gMDSCs expressing PD-L1 (Fig. 3c). However, more studies are required to determine the exact role of PD-L1 in MDSC mediated immunosuppression.

CD39 was analyzed as an additional marker associated with MDSC activation and suppression. During FV infection the expression of CD39 on MDSCs is high in both naïve and infected animals (Fig. 3a). CD39 is an essential molecule involved in an adenosine metabolism. First CD39 metabolizes ADP/ATP to AMP, which is followed by the conversion of AMP to adenosine. This action shifts an AMP-driven proinflammatory microenvironment to anti-inflammatory conditions driven by adenosine [84]. Interestingly, CD39 expression and function were linked to immunosuppression in various diseases models [85–87]. Adenosine metabolism has a great influence on T cells, macrophages, neutrophils. Prinicipally Tregs seem to mediate suppression due to CD39 function. During HIV infection Tregs show elevated CD39 expression which has been positively correlated with a disease progression [88]. Moreover, Tregs from HIV infected patients expressed higher levels of CD39, and effector T cells from these patients showed a higher sensitivity to adenosine in vitro [87, 89]. In our work we observed a decreased MDSC suppression of CD8^+^ T cells in the absence of CD39 (Fig. 3c). These data identify CD39 as a possible mechanism of MDSC mediated suppression. The function of CD39 on MDSCs was previously examined in an ovarian cancer model [90]. Ryzhov et al. [11] identified TGF-β as a regulator of CD39 expression on MDSCs. In colorectal cancer the expression of CD39 was also elevated on MDSC and these cells exhibited an increased inhibitory effect on T cells in comparison to cells from healthy donors [91].

The phenotypic characterization of MDSCs can be challenging, therefore their functional properties should be a focus of research. In the current work, MDSCs were functionally characterized by the expression of NOS2 and Arg1 and by specific blocking experiments in the T cell proliferation assay. Only the expression of Arg1, but not of NOS2, increased in gMDSCs after FV infection (Fig. 3a). These two important mechanisms of MDSC mediated immunosuppression are connected to ι-arginine metabolism. Both arginase and NO inhibitors were used in the current study, which confirmed that both pathways were partly involved in a gMDSC-mediated suppression of CD8^+^ T cell proliferation. Although the NO inhibitor was able to partially inhibit the gMDSC-mediated immunosuppression (Fig. 3b), our previous results showed no enhanced expression of NOS2 in gMDSCs. However, it is important to note that two additional enzymes are involved in the nitric oxide metabolism: NOS1 and NOS3 [92]. The expression and activity of NOS3 was reported to play a role in gMDSC suppression using a murine model of malignant melanoma [93]. Thus, NOS3 might also be involved in the suppression by virus-induced gMDSCs, a premise which yet has to be tested.

Different attempts to study the activity and relevance of MDSCs in vivo have been presented. First, transfer experiments were performed, in which freshly isolated, lipopolisaccharid (LPS) or IFNγ induced MDSCs were transferred into recipient mice. This transfer was shown to inhibit inflammation [94] and reduce CD8^+^ T cell responses in a melanoma model [95]. Another approach to study the function of MDSCs in vivo is a specific depletion of these cells. The most commonly used way of depletion is by administrating αLy6G or αGr1 antibodies into mice. This procedure allows the efficient depletion of all MDSCs (αGr1 antibody) or specifically of gMDSCs (αLy6G antibody).

In the current study, the MDSC function during an ongoing virus infection was investigated by using either αLy6G antibodies, which selectively deplete gMDSC, or 5FU, an anti-cancer drug known for selectively depleting all MDSCs [55]. Both αLy6G and 5FU administration resulted in the efficient depletion of MDSCs and led to an expansion of activated CD8^+^ T cells (Fig. 4c). Although both depletion attempts have proven to be MDSC specific, it is important to note that αLy6G is also used for the depletion of neutrophils, and that 5FU appears to be cytotoxic for other cell types in higher concentration. We therefore carefully analyzed several other important immune cell populations in our depletion experiments and verified that they were not affected (data not shown). Interestingly, the depletion of all MDSCs after treatment with 5FU had a stronger effect on CD8^+^ T cell responses than the depletion of only gMDSCs with anti-Ly6G treatment (Fig. 4c–e). Thus, mMDSCs, which were described to suppress lymphocyte responses in the murine AIDS model [25, 26], may also play role in FV infection in vivo. This data might be contradictory to the in vitro results, which showed no suppression of FV-specific T cells by mMDSCs. One possible explanation might be that gMDSCs directly affect antigen-specific CD8^+^ T cells, whereas mMDSC only indirectly suppress CD8+ T cell responses. One possible target of mMDSCs might be CD4^+^ T cells that then affect CD8^+^ T cell responses. Indeed, it has been shown that at day 14 after FV infection the populations of expanded CD4^+^ Tregs [96] and effector CD4^+^ T cells [97] have a strong impact on the quantity and the functionality of effector CD8^+^ T cells. It is known that MDSCs influence the expansion of Tregs [98] and CD4^+^ T cells [99] and thus have an indirect effect on the population of effector CD8^+^ T cells. In our in vitro assay system only DC and antigen-specific CD8^+^ T cells, but no CD4^+^ T cells were present. Thus, the in vitro model did not fully reflect the more complex situation in vivo. The influence of FV expanded MDSCs on different subpopulations of lymphocytes needs to be characterized in future studies.

Our data indicate in an in vivo approach that MDSC can indeed inhibit virus-specific T cell responses and interfere with virus immune control during an acute infection. This T cell suppression might be involved in the establishment or maintenance of viral chronicity.

## Conclusions

Overall, this work demonstrates the important regulatory role of expanded MDSCs during the late phase of acute FV infection. The suppressive effect on CD8^+^ T cells was predominantly mediated by gMDSCs, but mMDSCs may also contribute in vivo. Activated gMDSCs produced arginase 1 (Arg1), expressing the inhibitory ligand PD-L1 as well as the ATP dephosphorylating enzyme CD39 on their cell surface. All these effector molecules of MDSCs were involved in dampening virus-specific CD8^+^ T cell responses in vitro. Moreover, the in vivo depletion of MDSCs resulted in augmented virus-specific cytotoxic CD8^+^ T cell responses which correlated with a reduction in viral loads. Thus, our results demonstrate an inhibitory role of gMDSCs in FV infection and suggest these cells as a possible target for an immunomodulatory therapy of retroviral infections.

## Methods

### Mice

Inbred C57BL/6 (B6) mice were maintained under pathogen free conditions. Experiments were performed using B6 mice and were obtained from Charles River Laboratories. The relevant FV resistance genotype of B6 mice is H-2b/b, Fv1b/b, FV2r/r, Rfv3r/r. PD-L1 KO (B7-H1-KO) mice were originally generated by Lieping Chen [100]. CD39 KO mice on B6 background were originally produced by Simon C. Robson [101]. B6-background DbGagL TCR transgenic mice, where more than 90% of CD8^+^ T cells contained a TCR specific for DbGagL FV epitope [46, 47]. All mice were females between 8 and 16 weeks old at day 0 of the experiments.

### Virus and viral infection

The FV stock used for these experiments was a FV complex containing B-tropic Friend murine leukemia helper virus (F-MuLV) and polycythemia-inducing spleen focus forming virus, and was free of lactate dehydrogenase-elevating virus [102]. The stock was prepared as a 10% spleen cell homogenate from BALB/c mice infected 14 days previously with 3000 spleen focus-forming units of non-cloned virus stock. The virus was injected into experimental mice intravenously with 0.3 ml of PBS with 20,000 spleen focus forming units of FV.

### In vivo cell depletion

C57BL/6 mice were infected with FV. For the depletion of MDSC, 10 mg/kg body mass 5-Fluorouracil (5FU) (Sigma-Aldrich) was administrated i.p. 4 days prior sacrifice. To deplete gMDSCs, 100 µg of anti-Ly6G antibody (Clone 1A8) (BioXCell) or control ratg IgG antibody (BioXCell) was administrated every third day for 3 times intraperitoneal.

### Infectious center assay

Infectious centre (IC) assay was performed to determine the viral loads in infected organs. Shortly, tenfold dilutions of single-cell suspensions from the organ of interest were incubated with *Mus dunnis* cells for 3 days. After this time the cells were fixed with ethanol, stained with F-MuLV envelope-specific monoclonal antibody 720 and developed with peroxidase-conjugated goat anti-mouse antibody and aminoethylcarbazol to detect foci [103].

### Cell surface and intracellular staining by flow cytometry

The antibodies used for cell surface staining were obtained from eBioscience or BioLegend, anti-Ly6G (1A8), anti-Ly6C (HK1.4), anti-Gr1 (RB6-8C5), anti-CD11b (M1/70), anti CD19 (eBio1D3), anti-CD3 (17A2), anti-CD39 (24DMS1) anti-PD-L1 (MIH5), anti-CD80 (16-10A1), anti-CD8 (53-6.7), anti-CD43 (1B11), anti-NK1.1(PK136) anti-Ter119 (Ter-119) and FC block anti-mouse CD16/CD32 [91]. Intracellular Granzym B (monoclonal anti-human granzyme B (GB11) antibody Invitrogen) staining was performed as previously described [96]. Intracellular expression of Ki67 (SolA15), Arg1 (R&D Systems) and NOS2 (CXNFT) was detected using a Foxp3 staining kit (eBioscience). Dead cells for cell-surface and intracellular staining were excluded with use of Fixable Viability Dye eFluor 780 (FVD) (eBioscience). Data were acquired on a LSR II flow cytometer (Becton–Dickinson) from 300,000 to 500,000 lymphocyte-gated events per sample. Analyses were done using FlowJo (Treestar).

### Tetramer staining

To detect D^b^GagL-specific CD8^+^ T cells, splenocytes were stained with PE labelled MHC class I H2-D^b^ (Beckman Coulter, Marseille, France) tetramers, which are specific for FV GagL peptide [47, 104] as described previously described [96].

### In vitro suppression assay

To examine the influence of MDSCs in vitro on both the proliferation and function of CD8^+^ T cells, a standard in vitro immunosuppression assay was modified as described for the characterisation of the suppressive function of regulatory T cells. Murine bone marrow dendritic cells were generated as previously described and incubated with DbGagL peptide (5 µg/ml) in RPMI containing 10% FBS, 2 mM l-glutamine, 50 µM 2-ME and 100 U/ml each penicillin and streptomycin at for 60 min at 37 °C. FV-specific TCR Tg CD8^+^ T cells were isolated from the spleens of DBGagL TCR Tg mice by positive selection using magnetic bead separation according to the manufacturer’s instruction (Miltenyi Biotec), and then labelled with Violet Tracer dye (Life Technologies). Ly6G^high^ gMDSCs and Ly6G^low/−m^ MDSCs were isolated from the spleens dissected from B6, PD-L1 KO or CD39 KO mice by positive selection using magnetic bead separation according to the manufacturer’s protocol. (Miltenyi Biotec) For the induction of T cell proliferation, 1 × 10^5^ of peptide-pulsed DCs and 5 × 10^5^ TCR Tg CD8^+^ T cells were co-cultured on a flat-bottom 96-well plate in AIM-V (Life Technologies) containing 10% FBS. MDSCs were added to the culture simultaneously with CD8^+^ T cells. After 72 h cells were stained for CD8, fixed, permeabilized and stained for intracellular GzmB as described above. Additionally, for experiments that examined the effect of NO and arginase, the assay described above was implemented for the characterisation of gMDSCs function in vitro with the use of inhibitors. Besides bead-isolated CD8^+^ T cells from naïve TCRtg mice, peptide loaded DCs and Violet Tracer stained gMDSCs, an arginase inhibitor 0.5 mM nor-NOHA (NW-hydroxyl-nor-l-arginine) (Cayman Chemical) and NO inhibitor 0.5 mM L-NMMA (NG-Methyl-l-arginine acetate salt) (Sigma-Aldrich) were added at the beginning of the culture.

### Statistical analysis

Statistics comparing the two groups were done using the unpaired t test (GraphPad Prism software; GraphPad Software INC., San Diego, USA). When more than two groups were compared, a Dunn test with the Benjamini–Hochberg correction for multiple testing was performed (R-package dunn.test, version 1.3.4).
